# Ecological risk assessment of heavy metals in salt-affected soils in the Natura 2000 area (Ciechocinek, north-central Poland)

**DOI:** 10.1007/s11356-017-0323-5

**Published:** 2017-09-30

**Authors:** Agata Bartkowiak, Joanna Lemanowicz, Piotr Hulisz

**Affiliations:** 1 Department of Soil Science and Soil Protection, Faculty of Agriculture and Biotechnology, UTP University of Science and Technology, 6 Bernardyńska Street, 85-029 Bydgoszcz, Poland; 2 Sub-Department of Biochemistry, Faculty of Agriculture and Biotechnology, UTP University of Science and Technology, 6 Bernardyńska Street, 85-029 Bydgoszcz, Poland; 3 Department of Soil Science and Landscape Management, Faculty of Earth Sciences, Nicolaus Copernicus University, 1 Lwowska Street, 87-100 Toruń, Poland

**Keywords:** Enzymes, Halophytes, Heavy metals, Natura 2000, Soil salinity

## Abstract

This paper aimed to evaluate the ecological risk posed by the accumulation of heavy metals in the salt-affected soils of the habitat covered by the EU Natura 2000 program in relation to the activity of soil redox enzymes. The research was carried out in the halophyte reserve in Ciechocinek (north-central Poland) which is a very specific habitat as it undergoes a long-term human impact related to both the operation of the medical spa town and the agricultural use of soils in the adjacent areas. The obtained results showed that the content of Zn, Cu, Pb, and Cd in the studied soils exceeded the Polish standards. Based on the obtained data and statistical analysis, it was found that metals may come from two different sources: emission from household boiler rooms (Pb, Cd) and corroded brine sewage pipeline (Zn, Cu).They are characterized by limited mobility due to alkaline environment and strong sorption properties of the clay fraction and organic matter. The correlation analysis indicates that the dehydrogenase activities were negatively correlated with soil electrical conductivity (EC_1:5_) (*r* = − 0.665, *P* < 0.05). Taking into account the protective status of the area, it is difficult to indicate definitely the solution concerning the land management. However, according to the authors, one should pay special attention to a possibility of using halophytes which occur within the reserve for phytoremediation.

## Introduction

The Natura 2000 conservation area network has been established by the EU to preserve the most important and endangered habitats and species across Europe. Such areas show a very high susceptibility to unfavorable effect of human activity (Macedo-Sousa et al. 2009; Schröder et al. 2010; Tsiafouli et al. 2013). It comes mostly from the high variation in objects of protection and local environmental conditions within respective areas. Therefore, it is necessary to determine the sensitivity of each protected species and natural habitat to possible threats, including, e.g., the development of settlement network, emissions of pollution to the atmosphere, waters, and soils, excessive exploitation of natural resources (including deforestation), and excessive tourism (Pröbstl 2003; Jankowska-Huflejt 2006; Kopeć et al. 2016; Skordas et al. 2015). A negative reaction of the habitat to the effect of those threats can lead to its total degradation, resulting in a loss of the qualities the protection has been established for.

The accumulation of heavy metals in soils, as one of the key threats for Natura 2000 areas, is closely connected with intensive urbanization and industrialization. Heavy metals are natural, nondegradable substances and, as such, they are not broken down in the environment (Kabata-Pendias and Pendias 2001). The content of heavy metals in soils can originate from natural pedogeochemical properties, anthropogenic sources, or a mixture of these two sources. The ratios of these fractions vary widely depending on the type of substances and soil, land use, and the nature and extent of external impacts (Krishna and Govil 2007; Shakeri et al. 2009; Hu et al. 2013; Su et al. 2014). Soil contamination with heavy metals is a major problem as it leads to a negative effect on soil characteristics and the limitation of productive and environmental functions (Uzarowicz 2011; Vacca et al. 2012; Paz-Ferreiro et al. 2014; Kennou et al. 2015; Roy and McDonald 2015).The phytoavailability of such elements depends on soil properties, i.e., pH, cation exchange capacity, organic matter content, redox conditions, and salinity level. For example, a high chloride concentration in soil can have an effective role in increasing solubility of cadmium (Norvell et al. 2000; Weggler et al. 2004; Usman et al. 2005).

Monitoring of soils in protected areas under the Natura 2000 network is essential; it requires both comprehensive physicochemical and microbiological research. Soil enzymes are active in metabolism and catalyzed the processes related to matter processing and energy cycling in the soil environment (Orczewska et al. 2012; Siwik-Ziomek and Lemanowicz 2014). Many of them (for example, dehydrogenases, phosphatases, catalase, protease, and urease) have been proposed as indicators for measuring the degree of soil degradation. The sensitivity to small changes depends on a number of factors, e.g., flora (Bielińska and Gruszecki 2010; Lemanowicz and Bartkowiak 2013), soil type (Bartkowiak and Lemanowicz 2014), and soil profile depth (Lemanowicz and Krzyżaniak 2015). The applicable literature shows that mineral xenobiotics at large amounts inhibit the activity of soil enzymes (Lee et al. 2009; Wyszkowska et al. 2013).

However, there is little recognition of the problem of the effect of the accumulation of heavy metals on the enzymatic activity of soils under saline stress. For that reason, the aim of this paper was to evaluate the ecological risk posed by the accumulation of heavy metals in the salt-affected soils of the habitat covered by the EU Natura 2000 program in relation to the activity of soil redox enzymes. The research involved the halophyte reserve in the small Polish town (Ciechocinek; north-central Poland). It was assumed that long-term human impact related to both the operation of the medical spa town and the agricultural land use in the adjacent areas can affect the relationships between enzymatic activity and other soil properties. The research results can be useful for monitoring of the human-transformed habitats, e.g., in terms of developing new protection recommendations.

## Materials and methods

### Study area

**Fig. 1:**
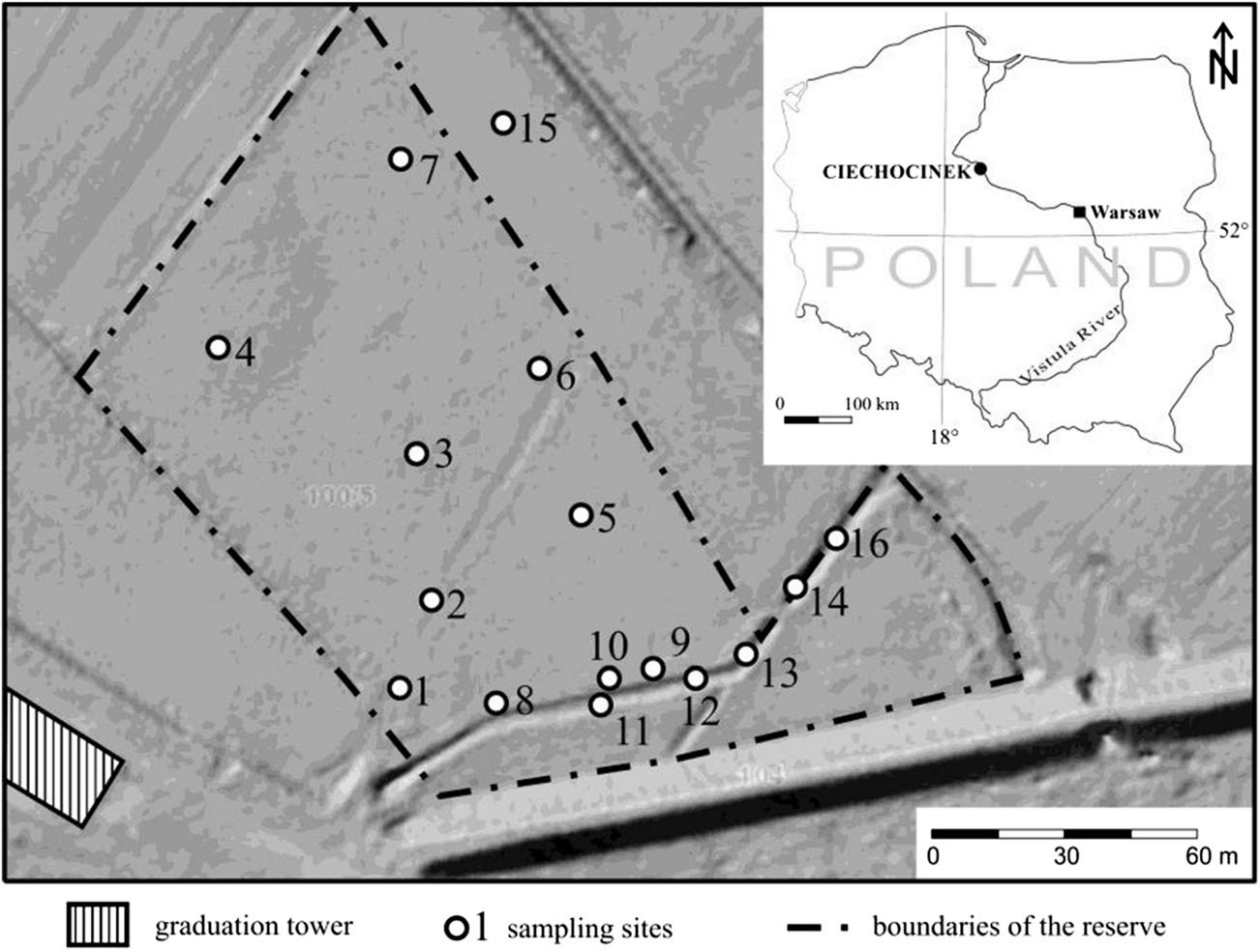
Localization of the study area

“Ciechocinek” halophyte reserve (1.88 ha), established in 1963, is one of the three reserves of halophytes in Poland. It is located in the Vistula river valley, adjacent to the graduation towers of the Ciechocinek medical spa (52°52’ N; 18°46’ E; Fig. 1). Wooden constructions from the nineteenth century, unique in Europe, were built in order to concentrate the brine in the process of salt production. Wooden graduation towers built according to the project of Stanisław Staszic have ca. 2 km in length and ca. 15 m in height. Oak balks constitute their base, whereas frames are built of pine wood. The interiors of these buildings are filled with brushwood of blackthorn (*Prunus padus* L.) (Gugnacka-Fiedor 2011). Those constructions are unique in Europe wooden structures filled up with blackthorn branches used for salt production process. The brine is condensed as a result of exposure to sunlight and wind and then transported by pipeline to the saltwork.

In the past, meadows in the Vistula River valley (including the reserve) were supplied by groundwater in contact with the Zechstein salt rock deposits. As a result of the valley irrigation and drainage, mostly in the 1950s and 1960s, the groundwater level decreased by more than 1 m. This action has contributed to an almost complete disappearance of halophytes (Wilkoń-Michalska 1962, 1970). Today, little areas of salt-affected soils exist only thanks to the supply with saline sewage from the medical spa town (also from the graduation towers), and thus, the communities of halophytes with *Salicornia europaea*, *Aster tripolium*, *Puccinellia distans* and *Spergularia salin* occur only within the ditch discharging saline waters, being the south-eastern border of the reserve (Warot and Nienartowicz 2001; Fig. 1). The area of the reserve is mowed and partially grazed. In December 2008, due to its unique character of halophytes on inland stands in Europe, the Special Area of Conservation Natura 2000 PLH 040019 Ciechocinek has been created. It also includes the area of halophytes reserve and the area of the neighboring agricultural land which in the 1960s was the area of salt meadows and the graduation tower (Fig. 1). The latter is a stand of halophytes which could again occupy the area of the reserve after introducing new protection rules (revitalization). The studied soils were formed on the alluvial sediments. According to the international soil classification WRB (IUSS Working Group WRB 2015), most of them can be referred to as Fluvic Gleysols (Salic, Sodic).

### Sampling and analysis

The fieldwork was conducted in 2015. A total of 16 sampling sites were located in the meadow (1–7), along the drainage ditch (8–14 and 16), and in the arable field (15). Soil samples were collected from the 0–20-cm depth in each site.

Before analytical processing, soil materials were air-dried at room temperature, disaggregated, homogenized, and sieved through a 2-mm mesh. The following analyses were performed for the soil samples: grain-size distribution by the laser diffraction method applying the Mastersizer MS 2000 analyzer, pH in H_2_O (PN-ISO 10390: 1997) and pH in 1 M KCl potentiometrically (PN-ISO 10390: 1997), the total organic carbon (OC) was determined with the TOC FORMACTSTM analyzer Primacs (provided by Skalar), electrical conductivity of 1:5 soil-water extract (EC_1:5_) using the conductometric method (van Reeuwijk 2002), and the content of selected heavy metals (Pb, Cu, Ni, Cd, and Zn) by atomic absorption spectrophotometry (AAS) after extraction of samples with a mixture of acids HF+HClO_4_ (Crock and Severson 1980).

Sixteen field-moist samples were sieved (2-mm mesh) and stored in a box at 4 °C for 2 days to stabilize the microbial activity and analyzed for selected redox: dehydrogenase and catalase activity within 1 week (Alef 1995; Alef and Nannipieri 1995). The activity of dehydrogenases (DEH) in soil was determined according to the Thalmann method (1968), after sample incubation with 2,3,5-triphenyl-tetrazolium chloride and a measurement of triphenylformazan (TPF) absorbance at 546 nm, and it was expressed in milligrams of TPF kg^−1^ 24 h^−1^. The activity of catalase (CAT) was assayed with the Johnson and Temple method (1964) with 0.3% hydrogen peroxide solution as a substrate. The residual H_2_O_2_ was determined using titration with 0.02 M KMnO_4_ under acidic conditions.

### Data analysis

Due to a considerable influence of anthropogenic transformations, the soils were evaluated using the enrichment factor percentage for heavy metal concentrations according to the following equation (Zonta et al. 1994; Loska and Weichula 2003):

$$ \%\mathrm{EF}=C-{C}_{\mathrm{min}}/\left({C}_{\mathrm{max}}-{C}_{\mathrm{min}}\right)\times 100 $$

where *C*—mean total concentration in soil, *C*_min_—minimum concentration, and *C*_max_—maximum concentration.

The enrichment factor (EF) can give an insight into differentiating an anthropogenic source from a natural origin. Five contamination categories are recognized based on the enrichment factor, where EF < 2 stands for deficiency to minimal enrichment, EF 2–5—for moderate enrichment, EF 5–20—for significant enrichment, EF 20–40 is very high enrichment, and EF > 40 is extremely high enrichment (Sutherland 2000).

With the values of the contents of heavy metals recorded in the soils, two geochemical parameters evaluating the anthropogenic effect on the soil environment were calculated.

(i) Contamination factor:

$$ \left(\mathrm{CF}\right)-\mathrm{CF}=C/{C}_n $$

where *C*—the mean content of metals from at least 16 sampling sites and *C*_*n*_—geochemical background.

(ii) Contamination degree (*C*_deg_) of the ecosystem as the sum of CF for all the metal yields studied.

As a geochemical background, the following values were adopted: Zn—19.0 mg kg^−1^, Cu—4.0 mg kg^−1^, Pb—7.1 mg kg^−1^, Ni—6.5 mg kg^−1^, and Cd—0.13 mg kg^−1^ (Czarnowska 1996).

Based on enzymatic activities of the samples, Biological Index of Fertility (BIF) was calculated according to Stefanic et al. (1984):

$$ \mathrm{BIF}=\left(1.5\ \mathrm{DEH}+100\ k\mathrm{CAT}\right)/2, $$

where *k* is a factor of proportionality equal to 0.01.

Basic statistics were used to study tendencies (mean, median) and the variability (standard deviation SD, coefficient of variation CV, minimum and maximum) of the sample population.

The coefficient of variation of the parameters analyzed was calculated as follows:

$$ \mathrm{CV}\%=\left(\mathrm{SD}/\mathrm{mean}\right)\times 100 $$

where СV—coefficient of variation (%), SD—standard deviation, mean—arithmetic mean.

The values, where 0–15%, 16–35%, and > 36%, indicate low, moderate, or high variability, respectively (Wilding 1985).

This paper presents the arithmetic means of the results from three replications. Besides, the results of the analyses of the features investigated were exposed to the analysis of simple correlation (*p* < 0.05 and *p* < 0.01), which determined the degree of dependence between respective parameters. In this study, physicochemical and chemical soil properties (pH, EC_1:5_, OC and heavy metals content, and enzymes activities) were analyzed applying the multivariate analyses (cluster analysis CA) with Ward’s method (1963). The result of hierarchical cluster analysis has been shown in a form of the dendrogram. Moreover, the principal component analysis (PCA) was used. The first two principal components (PC1 and PC2) were selected to determine which soil properties differentiate respective sampling sites. All the analyses were made using “Statistica 9.0 for Windows Pl” package.

## Results and discussion

### Basic soil properties and salinity

Due to Poland’s geographical location, the occurrence of salt-affected soils is conditioned only by the influence of waters with a high mineral content (of natural or anthropogenic origin). This process concerns mostly mineral-organic and organic soils which are characterized by lack of visible salinity features in their morphology. Therefore, the salinization may be considered as secondary to other soil forming processes (Hulisz et al. 2010, 2016).

The results demonstrated a high spatial variability of most basic soil characteristics (Table 1). According to the USDA classification (2006), four textural classes were distinguished: loamy sand (samples 1–3), sandy loam (samples 4, 8, 12, 13, and 16), silty loam (samples 5–7, 9–11, and 14), and loam (sample 15). The content of clay fraction (< 0.002 mm) ranged from 1 to 15% (CV—68%). The content of the organic carbon (OC) in soil ranged from 13.4 to 124 g kg^−1^. The spatial variation of that element (CV—64%) can be due to the effect of local environmental conditions, namely primary processes of the accumulation of river sediments, long-term exposure of those soils to greater moisture than today (namely before irrigation and drainage in the 1950s and 1960s), sodding, and mud accumulation in the ditch. Electrical conductivity of 1:5 soil-water extract (EC_1:5_) varied from 1.89 to 14.5 dS m^−1^ (mean 6.68 ± 5.06 dS m^−1^, Table 1). The highest level of salinity was reported for the soils located along the ditch discharging saline sewage from the medical spa town and brine from the graduation tower (samples 8–14 and 16). According to the formula proposed by Rengasamy (2006), which facilitates converting the EC_1:5_ results to saturated extract, half of the soil samples analyzed were classified as strongly saline (EC_e_ > 16 dS m^−1^) (Jackson 1958). The field studies demonstrated the occurrence of poorly permeable formations (clays) in the subsoils, especially in the area adjacent to the ditch. Therefore, salt migration at greater depth is limited here and evaporation increases soil salinity. On the other hand, the effect of salt aerosols from the graduation tower area on soil properties cannot be excluded. This was particularly evident in less saline soils located further from the ditch area (samples 1–7 and 15). As reported by Czajka et al. (2006), the average concentration of Cl^−^ ions in aerosol around the graduation towers at Ciechocinek is between 0.24 and 9.83 mg m^−3^.

**Table 1:** Basic properties of the studied soils

Sample no.	pH		EC_1:5_ (dS m^−1^)	OC (g kg^−1^)	Percentage share of fraction		
	H_2_O	KCl			Sand*	Silt	Clay
1	5.5	4.6	2.47	33.8	83	16	1
2	6.8	6.4	2.54	21.7	81	18	1
3	5.8	4.9	2.31	13.4	76	22	2
4	5.5	4.3	1.89	25.4	63	34	3
5	5.7	4.7	2.59	70.6	32	60	8
6	5.4	3.9	2.11	69.8	41	51	8
7	5.5	4.8	2.68	34.8	21	66	13
8	7.5	7.4	9.25	38.9	61	34	5
9	7.6	7.4	10.2	36.3	41	53	6
10	7.5	7.3	10.9	41.2	45	50	5
11	7.7	7.3	9.89	60.5	30	55	15
12	7.6	7.8	14.5	24.6	64	33	3
13	7.1	6.4	13.6	124	52	43	5
14	7.3	7.6	4.75	19.6	17	72	11
15	7.5	7.2	2.01	29.8	52	40	8
16	7.6	7.4	15.2	40.0	48	47	5
Mean	6.7	6.2	6.68	42.7	50	43	6
Median	7.2	6.8	3.72	35.5	50	45	5
Min	5.4	3.9	1.89	13.4	17	16	1
Max	7.7	7.8	14.5	124	83	72	15
SD	1.0	1.4	5.06	27.4	20	17	4
CV%	14	23	76	64.0	40	38	68

*Sand [2.0–0.05 mm], Silt [0.05–0.002 mm], Clay [< 0.002 mm]

The ranges of pH-H_2_O and pH-KCl for the soil samples analyzed were 5.4–7.7 and 3.9–7.8, respectively. In general, the pH values measured in H_2_O for non-saline soils and with a low content of calcium carbonate (also non-limed) are from about 1 to 1.5 units higher than those measured in KCl (Bednarek et al. 2004). Such dependence was observed only for a few samples (Table 1). In soils containing neutral salts, e.g., NaCl or limed, the soil reaction is usually neutral or slightly alkaline, and both values can be very similar (Abrol et al. 1988; Piernik et al. 2015). Such properties were recorded for soil samples located along the ditch (samples 8–14 and 16), as well as in arable field (15). A significant relationship between EC_1:5_ and pH values was also confirmed by high values of the correlation coefficient (Table 4).

### Heavy metal content and its relation to other soil parameters

Soil contamination with heavy metals is currently considered as one of the most serious environmental problems due to heavy metal persistence and toxicity, having a great impact as the development of areas without soil in good condition is difficult (Salazar and Pignata 2014). Concentrations of heavy metals in soils can result from natural or anthropogenic factors, with the latter being most common. Metals are usually nondegradable and become toxic if they exceed their threshold level, which poses a threat to biological life. Heavy metals may be bound or sorbed by particular natural substances, which may increase or decrease mobility (Dube et al. 2001). The total content of the analyzed form of heavy metals varied widely: zinc from 23.2 to 420 mg kg^−1^, copper from 7.48 to 432 mg kg^−1^, nickel from 0.44 to 21.2 mg kg^−1^, lead from 10.9 to 531 mg kg^−1^, and cadmium from 0.00 to 3.49 mg kg^−1^. A high spatial variability of the results was confirmed by the calculated coefficients (Table 2). The sources and distribution of potentially toxic elements like Cu, Ni, Pb, Cd, and Cr in the urban environments have been widely studied (Madrid et al. 2002, 2006; Elik 2003). In the town of Ciechocinek, there are many structures, houses, old villas, and guest houses, which are heated in a traditional way. Therefore, a probable source of pollution are dusts from household boiler rooms. As reported by the Provincial Inspectorate of Environmental Protection in Bydgoszcz (http://www.wios.bydgoszcz.pl/), the admissible level of daily average PM10 (particulate matter <10 μm; 50 μg m^−3^) was exceeded 43 times during 2015 at a limit value of 35 times (Journal of Laws, item 1031, 2012).

**Table 2:** The content of total zinc, copper, nickel, lead, and cadmium and the activity of dehydrogenases and catalase in the studied soils

Sites	Zn	Cu	Ni	Pb	Cd	DEH (mg TPF kg^−1^ 24 h^−1^)	CAT (mg H_2_O_2_ kg^−1^ h^−1^)
	(mg kg^−1^)						
1	23.2	7.48	1.26	31.6	0.35	0.121	0.031
2	32.7	7.66	1.29	32.2	bdl	0.632	0.121
3	25.0	9.33	2.09	52.2	bdl	0.547	0.042
4	32.0	9.60	3.58	89.4	bdl	0.516	0.053
5	87.8	29.2	21.2	531	0.16	0.558	0.064
6	81.5	23.3	15.4	385	0.41	0.095	0.039
7	77.2	29.3	21.0	525	0.16	0.154	0.076
8	94.1	225	3.88	96.9	1.25	0.119	0.087
9	344	102	1.15	28.8	bdl	0.096	0.032
10	420	74.1	0.44	10.9	0.49	0.091	0.029
11	112	16.3	17.4	436	0.30	0.106	0.026
12	231	26.9	1.06	26.6	3.49	0.085	0.021
13	144	432	13.2	329	0.93	0.095	0.035
14	269	59.7	0.68	16.9	0.89	0.289	0.048
15	82.2	76.1	18.8	470	0.90	0.781	0.298
16	275	82.4	1.15	28.8	0.94	0.049	0.019
Mean	146	75.7	7.72	193	0.64	0.271	0.063
Median	90.9	29.2	2.83	70.8	0.38	0.120	0.045
Min	23.2	7.48	0.44	10.9	0.00	0.049	0.019
Max	420	432	21.2	531	3.49	0.781	0.298
SD	124	110	8.35	209	0.86	0.245	0.070
CV%	85	145	108	108	134	91	107

*bdl* below detection limit

The fallout from smokestacks can reach the residential areas depending on the prevailing wind directions. It is known that untreated or unfiltered emissions from most types of combustion and incineration facilities will also carry different trace metals, especially Cr, Ni, Cu, Zn, and Pb (Govil et al. 2008). Road transport is also a source of air pollution with carbon oxides, hydrocarbons, and lead compounds. According to Li et al. (2004), the concentration of Pb in urban soils reflects the significant degree of historical Pb contamination and the long half-life of Pb in soils. Low emissions are especially onerous in urban agglomerations and wherever the conditions are unfavorable to the spread of pollution, hence the importance of actions to limit it, e.g., by changing from coal boiler facilities into gas and oil heating. At the same time, the objective is to restrict heating houses with various kinds of waste which often, during the burning process, release considerable amounts of toxic substances. According to the Regulation of the Minister of Environment dated 9 September 2002 on standards for soil quality and land quality standards (Journal of Laws No 165, item 1359, 2002) and dated 1 September 2016 on assessment procedures for the land surface pollution (Journal of Laws item 1395, 2016), it was stated that the admissible contents of Zn, Cu, Pb, and Cd were exceeded in the studied soils (Table 2). High amounts of zinc (94.1–420 mg kg^−1^) and copper (16.3–432 mg kg^−1^) found in soils sampled along the ditch probably resulted from the corrosion of the brine pipes. It may cause some Cu and Zn emissions to the environment, which was confirmed by a significantly positive value of the coefficient of correlation between the content of zinc and electrical conductivity (Zn EC_1:5_ *r* = 0.668, *P* < 0.05), as well as the content of copper (Cu EC_1:5_ *r* = 0.509, *P* < 0.05—Table 4). Some physicochemical properties of soils, pH, texture, and organic carbon, are important parameters that control the accumulation and the availability of heavy metals in the soil environment (Dube et al. 2001; Weng et al. 2002; Ashworth and Allowy 2008; Shakeri et al. 2009). Organic matter can be a factor in releasing as well as immobilizing metals in soil. Such dependence can be confirmed by the analysis of correlation as showing significantly positive values of the coefficient of correlation between the content of copper and organic carbon (*r* = 0.688, *P* < 0.05), nickel and organic carbon (*r* = 0.510, *P* < 0.05), and lead and organic carbon (*r* = 0.510, *P* < 0.05) (Table 4). Moreover, the statistically significant relationships between the content of clay fraction and Pb and Ni as well as pH and Zn and Cd were found (Table 4).

### Degree of anthropogenic soil transformations

**Fig. 2:**
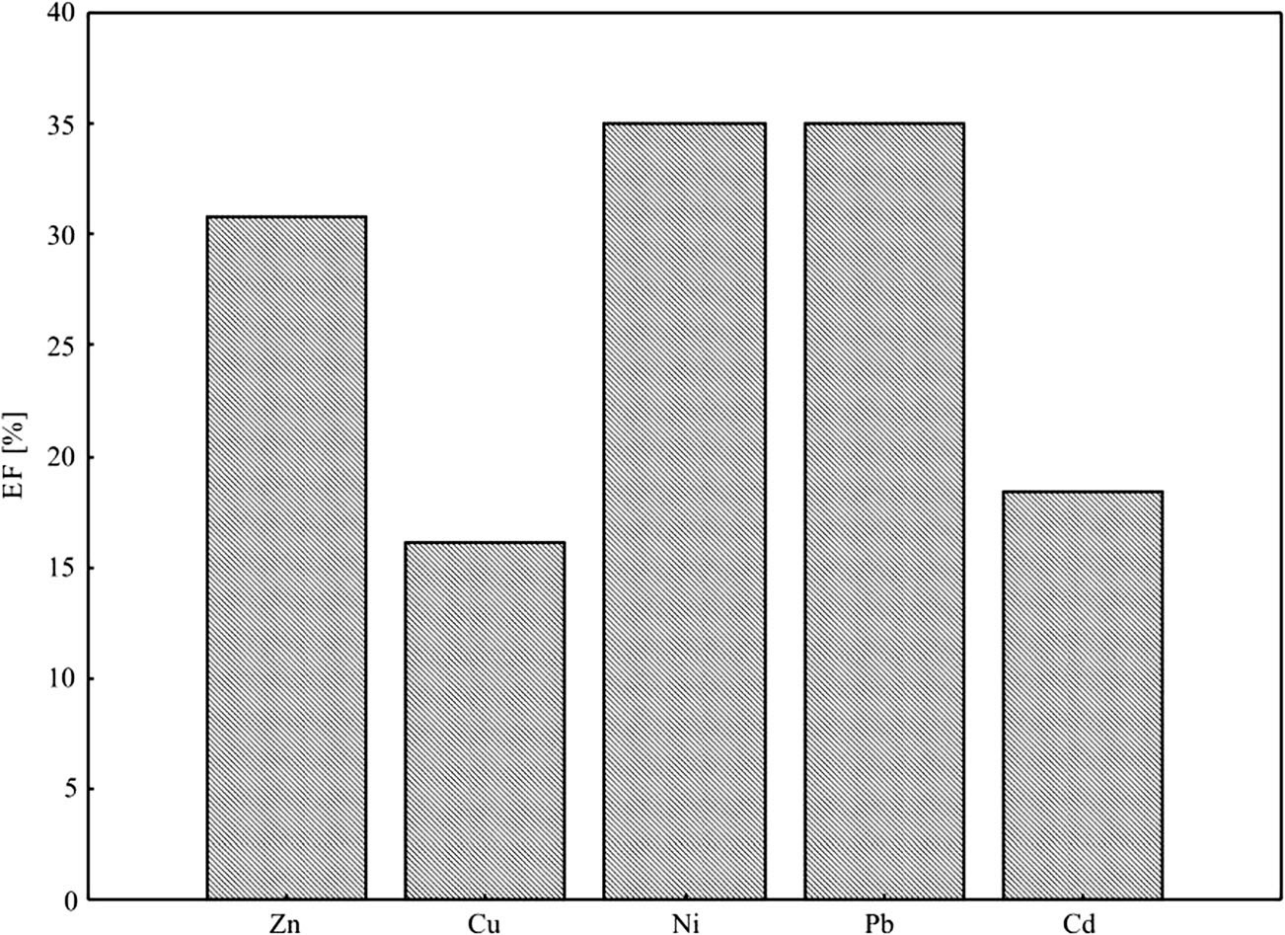
Enrichment factor

The enrichment factor is used to differentiate trace metals originating from human activities and those of natural sources. Basically, as the EF value increases, the contribution from non-crustal sources increases. The calculated values of the coefficient of pollution for the surface horizon of the soil in a given area (EF) point to a high pollution with copper (EF—16%) and cadmium (EF—18%), whereas zinc, nickel, and lead indicated extreme contamination of surface soil horizons (Fig. 2)*.* Literature reports on (Ali and Malik 2011; Atiemo et al. 2011; Ramachandra and Shwetmala 2009; Thorpe and Harrison 2008; Loska et al. 2004) increased EF values in urban soils are mostly due to industrial and road traffic emissions.

The contamination coefficient (CF) allowed for the classification of the soils to the appropriate group, depending on the geochemical background factor. The contamination factor or anthropogenic factor for each element was computed and the result is presented in Table 3. According to the criterion developed by Håkanson (1980), the soil from sampling sites 9–10, 12–14, and 16 demonstrated a very high value of the contamination factor of zinc; from sampling sites 5, 7–10, 12–16 with copper; from sampling sites 3–8, 11, 13, and 15 with lead; and from sampling sites 8, 12–16 with cadmium. The sum of contamination factors for all the metals examined indicates a considerable degree of contamination in soil. It is inferred that the contamination comes from the anthropogenic sources. The overall assessment of the soil pollution was tested based on the degree of contamination (*C*_deg_). According to Håkanson (1980), only for a single sampling site (no. 2) was there identified a low degree of contamination, sampling sites 1, 3, and 4 moderate, and in the others—high and very high (Table 3). The degree of contamination in soil samples is considerable and very high; there should be a thorough heavy metal monitoring in soil to prevent relative health hazards to man and livestock in the area (Loska et al. 2004; Bambara et al. 2015).

**Table 3:** Coefficients and degrees of soil contamination

Sites	CF					Cdeg	CF in Cdeg (%)				
	Zn	Cu	Ni	Pb	Cd		Zn	Cu	Ni	Pb	Cd
1	1.22	1.87	0.19	4.45	2.69	10.4	11.7	18.0	1.23	42.7	25.8
2	1.72	1.92	0.19	4.53	0	8.36	20.6	23.0	2.27	54.2	0
3	1.31	2.36	0.32	7.35	0	11.3	11.6	20.9	2.83	65.0	0
4	1.68	2.40	0.55	12.6	0	17.2	9.76	15.7	3.19	73.1	0
5	4.62	7.30	3.27	74.7	1.25	91.2	5.07	8.00	3.59	59.5	1.37
6	4.29	5.84	2.37	54.3	3.18	70.0	6.13	8.35	3.39	77.6	4.55
7	4.06	7.32	3.23	74.0	1.25	89.9	4.52	8.15	3.59	82.4	1.39
8	4.95	56.3	0.59	13.6	9.61	85.1	5.81	66.2	0.69	16.0	11.3
9	18.1	25.6	0.17	4.05	0	47.8	37.9	53.5	0.36	8.47	0
10	22.1	18.5	0.06	1.54	3.75	46.0	48.1	40.3	0.13	3.35	8.16
11	5.88	4.08	2.68	61.4	2.30	76.3	7.71	5.35	3.21	80.4	3.01
12	12.1	6.72	0.16	3.74	26.8	49.6	24.5	13.6	0.32	7.54	54.1
13	7.58	108	2.02	46.3	7.11	171	4.43	63.2	1.18	27.1	4.16
14	14.1	14.9	0.10	2.38	6.83	38.4	36.9	38.9	0.26	6.20	17.8
15	4.33	19.0	2.89	66.2	6.92	99.4	4.36	19.2	2.91	66.6	6.96
16	14.5	20.6	0.17	4.05	7.21	47.0	30.8	43.9	0.36	8.63	15.4

*CF* contamination factor, *CF in C*_*deg*_ degree of contamination

The enzyme activities (catalase, dehydrogenases) showed fluctuating results in the present study (Table 2). The activities of catalase in soil ranged from 0.019 to 0.298 mg H_2_O_2_ kg^−1^ h^−1^ (with a mean value of 0.063 mg H_2_O_2_ kg^−1^ h^−1^) and dehydrogenases from 0.049 to 0.781 mg TPF kg^−1^ 24 h^−1^ (with a mean value of 0.271 mg TPF kg^−1^ 24 h^−1^). The highest activity of enzymes in soil was reported from site 15 (agricultural area)—CAT 0.298 mg H_2_O_2_ kg^−1^ h^−1^ and DEH 0.791 mg TPF kg^−1^ 24 h^−1^. Dehydrogenase activity showed a significant negative correlation with soil EC_1:5_ (*r* = −0.665, *P* < 0.05). Similarly, Batra and Manna (1997) report on a negative dependence between salinity and the activity of dehydrogenases (*r* = −0.767). Dehydrogenase is an intracellular enzyme, considered an indicator of the viable microbial activity in soils. The influence of soil EC on enzyme activities might involve the level of soil salt affecting enzyme configuration and formation of its activity center. The process of protein salting out occurs and so enzymes lose its biological activity. Similar results are reported by Siddikee et al. (2011) who studied the influence of varying degree of salinity-sodicity stress on enzyme (dehydrogenases, cellulase, glucosidase, protease, alkaline, and acid phosphatase) of coastal soils of the Yellow Sea, South Korea. The enzyme activity associated with salinization varied depending on the degree of salinization, as well as on the type of enzyme (Pan et al. 2013).There was recorded no negative dependence between the activity of catalase and pH, EC_1:5_, whereas Shi et al. (2008) report on soil catalase activity being closely related to soil EC and no significant relationship with other physicochemical properties. Catalase can split hydrogen peroxide into molecular oxygen and water and thus prevent cells from damage by reactive oxygen species. According to Jaspers and Kangasjärvi (2010) and Wu et al. (2012), catalase is an anti-oxidation enzyme, participating in the defense of the plants from abiotic and biotic factors triggering oxidation stress among plants, e.g., growing in saline soils. Catalase is active over a wide pH range and its activity does not drop until pH is below 3.5. In the samples under study, pH was high and ranged from 5.4 to 7.7 in H_2_O and 3.9 to 7.8 in 1 M KCl. According to Wilding (1985) classification, the CV values of soil dehydrogenase activities (91%) and catalase activity (106%) were high.

**Fig. 3:**
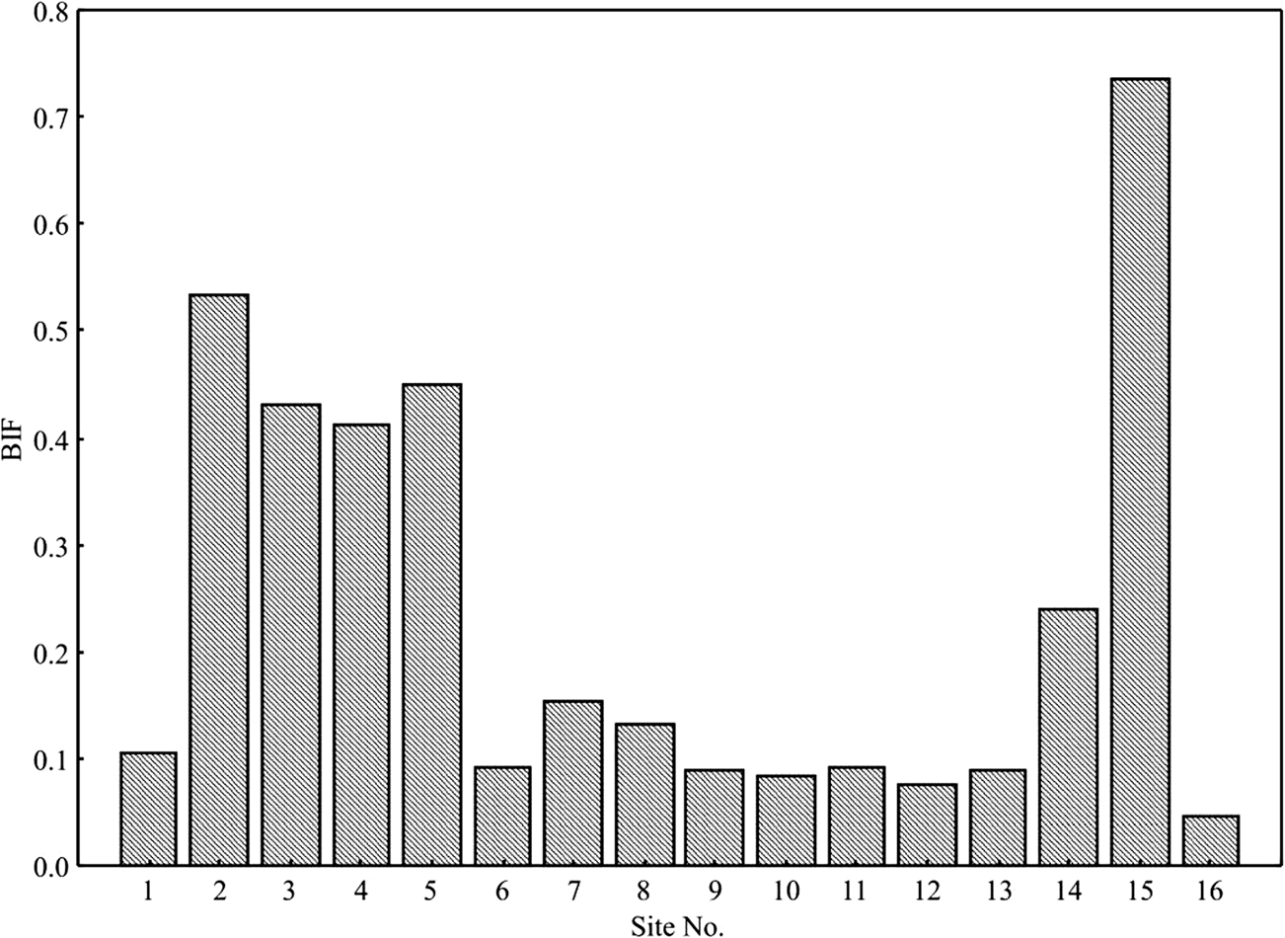
Value samples BIF (sites from 1 to 16)

The value of BIF (Stefanic et al. 1984) calculated based on the activity of dehydrogenases and catalase was highest (BIF 0.735) in the soil sampled from the arable field (site 15) (Fig. 3). In the soil sampled from the sites with the highest salinity (EC_1:5_), the value of the biochemical fertility index was lowest (BIF < 0.1) (sites 6, 9, 10, 11, 12, 13). A similar dependence was earlier reported by Telesiński (2012), while Saviozzi et al. (2011) report on BIF values not being considerably different from the control sample and the sample under study, indicating that such an index is unable to determine effects of salt on the biological activity of soil.

**Fig. 4:**
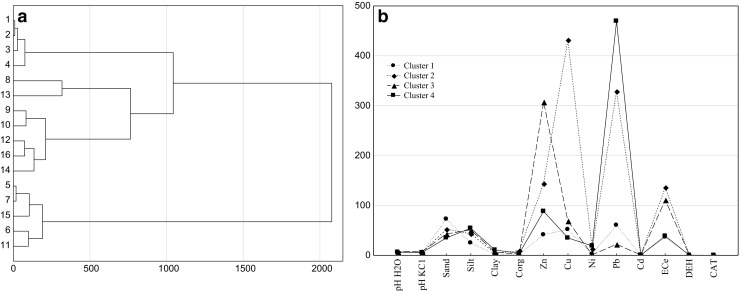
Similarity dendrogram for sampling sites (1–16) (**a**) and a graph *k* − means for the study properties (**b**)

Cluster analysis was used to identify the similarity of the groups between the samples from points grouping all 16 sampling sites into four significant clusters (Fig. 4a). Based on the data clustering with Ward’s method (1963), it was found that cluster 2 includes two soil samples (samples 8 and 13). Those sampling sites showed a higher copper content. Five soil samples (9, 10, 12, 14, and 16) were included in cluster 3 (Fig. 4a) due to a high zinc content and electrical conductivity (EC_1:5_) (Fig. 4b). Five soil samples (5, 6, 7, 11, and 15) fell within cluster 4 due to a high lead content and low EC_1:5_, the content of copper and sand. It is seen from the dendrogram that cluster 4 is characterized by the biggest Euclidean distance to the other clusters (high significance of clustering).

**Fig. 5:**
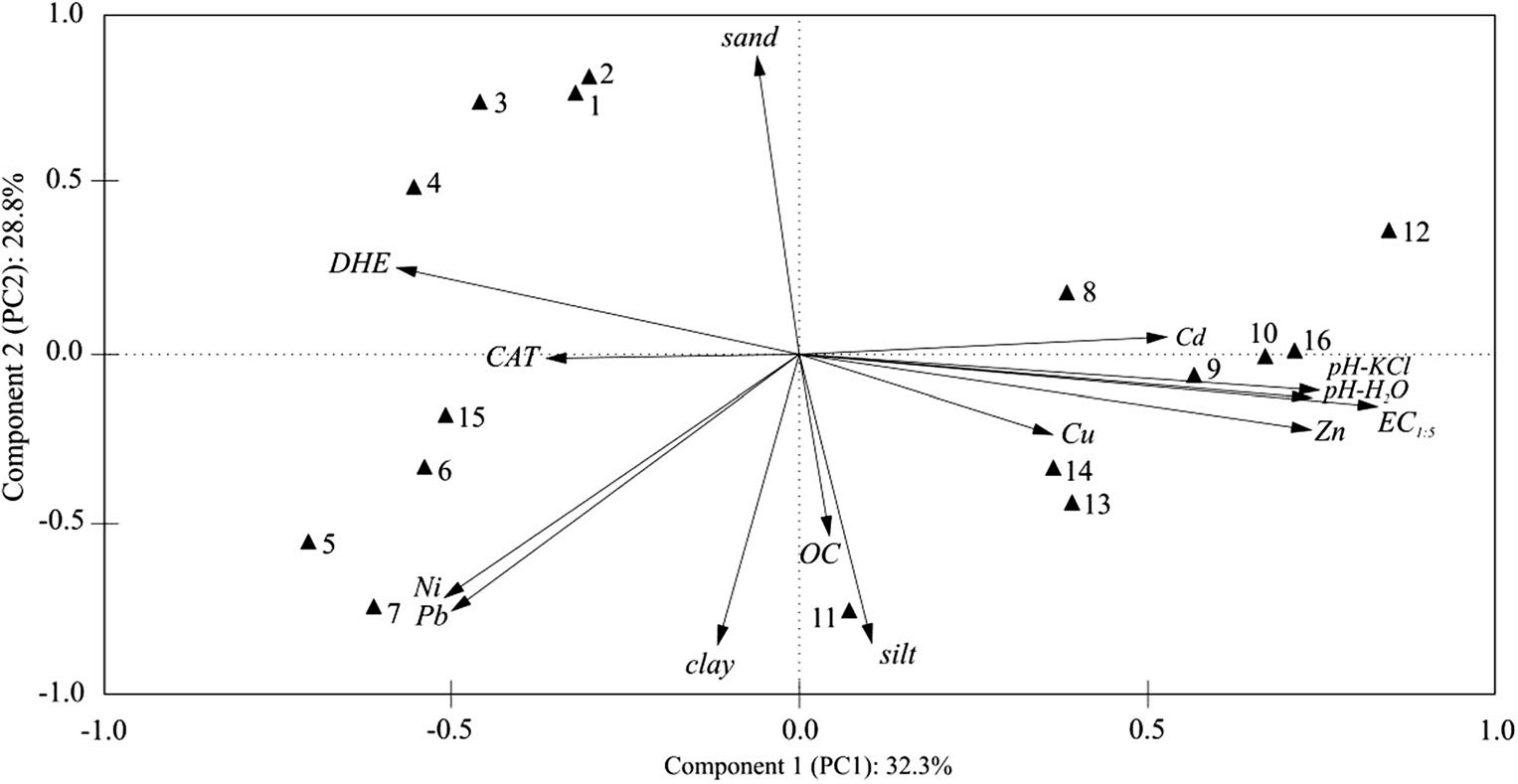
Configuration of variables in the system of the first two axes PC1 and PC2 of principal components

Table 5 displays the factor loadings, as well as the eigenvalues. Two principal components (PC1 and PC2) were extracted from the available data set that explained a total variance of approximately 61.02%. Factor 1 (PC1) is responsible for 32.26% of the total element variables and indicated a high correlation with pH in H_2_O (0.807) and KCl (0.818), the content of zinc (0.806), and EC_1:5_ (0.910). Factor 2 (PC2) is dominated by sand (0.909), silt (0.880), and clay (−0.885) and the contents of nickel (−0.740) and lead (−0.740). These two heavy metals may reflect the anthropogenic contamination in saline soil grasslands. The activity of dehydrogenases and catalase was negatively correlated with sites 8, 9, 10, 12, 13, and 16 (sites along the drainage ditch). The results of the analysis show that soil EC_1:5_, pH, and the content of zinc had a negative effect on the activity of soil enzymes, as indicated by the vectors along the axes of the coordinate system. Figure 5 shows correlations between the content of zinc and copper. Sampling sites 8, 9, 10, 12, 13, and 16 are located closer to the vectors of those variables, which proves that the soil at those sites is contaminated with Zn and Cu originated from corroded brine pipes.

### Proposal for land management

According to the results, most of the soils showed strong sorption properties of the clay fraction and organic matter (Tables 1 and 4). However, the properties of soils located along the ditch (samples 8–16) can significantly favor the limited mobility of heavy metals in alkaline environment **(**Brümmer and Herms 1983; Kabata-Pendias and Pendias 2001). However, some studies revealed that salinity can increase metal mobility (e.g., Hatje et al. 2003; Acosta et al. 2011). This is caused by both the complexation of salt anions with heavy metals and the competition between salt cations with heavy metal ions for sorption sites at the solid phase.

**Table 4:** Relationship between selected soil properties (*n* = 16; *P < 0.05*)

Variables		Equation	*r*
Dependent	Independent		
Total copper	Organic carbon	y = 42.6643 + 35.987x	0.688
Total nickel	Organic carbon	y = 0.10634 + 2.0257x	0.510
Total lead	Organic carbon	y = 26.5859 + 50.6427x	0.510
Total zinc	EC_1:5_	y = 36.6079 + 1.6333x	0.668
Total copper	EC_1:5_	y = 1.8252 + 1.1061x	0.509
Dehydrogenases	EC_1:5_	y = 0.4866–0.0032x	−0.665
Total nickel	Clay	y = −0.6293 + 1.3467x	0.678
Total lead	Clay	y = −15.7324 + 33.6666x	0.678
Total zinc	pH KCl	y = −214.9774 + 58.1292x	0.664
Total cadmium	pH KCl	y = −1.2889 + 0.3112x	0.510
EC_1:5_	pH H_2_O	y = −19.53 + 3.8975x	0.736
EC_1:5_	pH KCl	y = −8.9739 + 2.5226x	0.705

**Table 5:** Values of the two extracted factor loadings for 14 elements

Parameters	Component matrix	
	PC1	PC2
pH H_2_O	0.807*	−0.131
pH KCl	0.818*	−0.108
Sand	−0.066	0.909*
Silt	0.114	−0.880*
Clay	−0.128	−0.885*
OC	0.047	−0.553
Zn	0.806*	−0.229
Cu	0.399	−0.243
Ni	−0.558	−0.740*
Pb	−0.558	−0.740*
Cd	0.578	0.053
EC_1:5_DEH	0.910*	−0.158
CAT	−0.634	0.264
	−0.398	−0.012
Variation (%)	32.26	28.76
Eigenvalue	4.516	4.026

*Statistically significant

Taking into account the protective status of the area, it is difficult to show definitely a solution for land management. As mentioned earlier, in the near future, the revitalization of that reserve is planned; it is to facilitate the succession of halophytes beyond the zone around the ditch. With that in mind, according to the authors, one shall focus on a possibility of using native halophyte species for phytoremediation. As tolerant and resistant to an excess of toxic ions, mainly sodium and chloride, these plants may also be able to accumulate heavy metals (Manousaki and Kalogerakis 2011; Lutts and Lefevre 2015).These elements are mainly accumulated in the root, with some quantities translocated to the stems and leaves, especially in *Salicornia europaea* and *Aster tripolium*—halophytes commonly occurred in the study area (Lüttge 1975; Milić et al. 2012). As such, the plants would be ideal for phytostabilization rather than phytoextraction, which, however, requires more detailed research.

## Conclusions

The obtained results clearly demonstrated that due to the long-term human impact, the studied salt-affected soils were contaminated by heavy metals. Under the Regulation of the Minister of Environment of 9 September 2002 on standards for soil quality and land quality standards (Journal of Laws No 165, item 1359, 2002), it was found that the admissible contents of Zn, Cu, Pb, and Cd were exceeded in the study area. Based on the PCA analysis and EF index, it was revealed that Zn, Cu, Pb, Cd, and Ni were controlled by various anthropogenic activities (agricultural and human activities, vehicular emissions, etc.). The analysis of correlation confirmed the effect of a high salinity level, content of organic carbon, and the clay fraction on the content of heavy metals in soils. The BIF values facilitate monitoring the effects of salts on selected biological activity of soils. Effects of salinization on soil enzymes in the grassland habitat Natura 2000 in Ciechocinek (Poland) must be further investigated to provide rational management measures to promote long-term sustainability. A lack of inhibition of the enzymes as affected by heavy metals could have been due to the content of organic matter and clay fraction which bind enzymatic protein, protecting it from negative environmental factors. It is also known that heavy metals at little concentrations are activators of many enzymes. Bearing in mind the revitalization of the research area planned, one could also consider the applicability of some native halophytes to phytoremediation.
